# Quantitation of guanidine derivatives as representative persistent and mobile organic compounds in water: method development

**DOI:** 10.1007/s00216-023-04613-x

**Published:** 2023-02-28

**Authors:** Makiko Ichihara, Daichi Asakawa, Atsushi Yamamoto, Miki Sudo

**Affiliations:** 1 Osaka City Research Center of Environmental Science, 1-3-3 Nakamichi, Higashinari-Ku, Osaka, 537-0025 Japan; 2 Faculty of Environmental Studies, Tottori University of Environmental Studies, 1-1-1 Wakabadai-Kita, Tottori, Tottori 689-1111 Japan; 3 Department of Biological Resources Management, School of Environmental Science, The University of Shiga Prefecture, 2500 Hassaka-Cho, Hikone, Shiga 522-8533 Japan

**Keywords:** 1,3-Diphenylguanidine, Cyanoguanidine, Guanidine derivative, Persistent and mobile organic compounds (PMOCs), Analytical method development, Hydrophilic interaction liquid chromatography (HILIC)

## Abstract

Persistent and mobile organic compounds (PMOCs) are highly soluble in water, thereby posing a threat to water resource quality. Currently, there are no methods that can accurately quantify guanidine derivative PMOCs, other than 1,3-diphenylguanidine (DPG) and cyanoguanidine (CG), in aqueous media. In this study, we developed a quantitation method that combines solid-phase extraction and liquid chromatography (LC)-tandem mass spectrometry to detect seven guanidine derivatives in aquatic environments and applied it to environmental water samples. Five LC columns were examined, and among them, a hydrophilic interaction liquid chromatography column was chosen owing to its suitable instrument detection limit and retention factor. Method precision was assessed using seven replicate analyses of river water. The corresponding analyte recoveries ranged from 73 to 137% (coefficient of variation = 2.1–5.8%). DPG and CG were detected in ultrapure water samples at levels up to 0.69 and 150 ng L^−1^, respectively; DPG and CG levels up to 44 and 2600 ng L^−1^, respectively, were detected in lake water, river water, sewage effluent, and tap water sampled in Western Japan. This is the first reported detection of DPG in the surface water of Japan, revealing that DPG and CG are ubiquitous compounds in aquatic environments. Moreover, this is the first study to detect 1-(*o*-tolyl)biguanide and *N*,*N′′′*-1,6-hexanediylbis(*N′*-cyanoguanidine) in water. This study provides a foundation for further research on the distribution, fate, and emission source of these pollutants, which is critical to maintain high water quality and to determine regulatory limits for these pollutants.

**Graphical Abstract:**

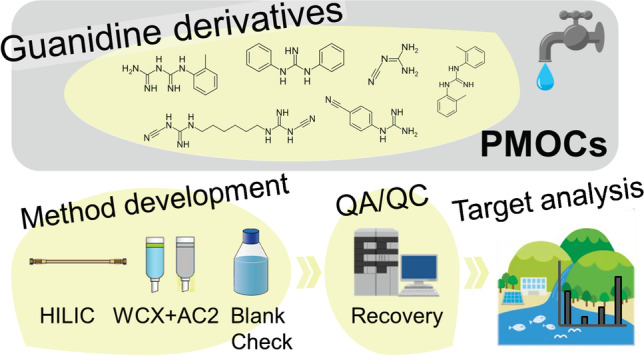

**Supplementary Information:**

The online version contains supplementary material available at 10.1007/s00216-023-04613-x.

## Introduction

Persistent and mobile organic compounds (PMOCs) are defined as highly polar compounds that persist in the environment [1]. They are difficult to remove after they enter an aquatic environment because they are highly water soluble and remain in water even after purification in treatment plants, as evidenced by the detection of these compounds in tap water [2]. The Scientific Committee on Health, Environmental and Emerging Risks (SCHEER) considers PMOCs an emerging threat to human health and the environment [3]. It equates the concerns regarding PMOCs to those of PBT (persistent, bioaccumulative, and toxic) substances. As PMOCs are PMT (persistent, mobile, and toxic) compounds, they pose an additional threat to humans in the form of contaminated drinking water [4, 5].

In recent studies, PMOCs have been ranked by priority to determine those substances that should be preferentially investigated [6, 7]. Arp et al. [6] identified ~ 2000 PMOCs (PMOC score = 4–5) among high-production-volume chemicals with registered REACH regulations in the EU, and Schulze et al. [7] used these data to compile a list of 936 high-priority PMOCs based on the amounts discharged into the environment. Furthermore, Schulze et al. [2] conducted a screening survey in an aquatic environment for 64 PMOCs from this list and prioritized those compounds requiring future investigation.

1,3-Diphenylguanidine (DPG) is a PMOC that is used as a vulcanization accelerator in rubber products such as tires [8, 9]. It was detected as one of the main leachables in lab-scale tire wear extraction experiments [10] and was classified as a tire-related chemical in source-related smart suspect screening in water [11]. In 2019, the manufacturing and import volume of DPG in Japan equaled 1000–2000 tons [12]. In view of its acute toxicity to aquatic organisms [13], DPG is classified as a PMT substance and has received considerable attention as an emerging PMOC, as exemplified by its detection in surface water [11, 14–23]. Similar to DPG, 1,3-di-*o*-tolylguanidine (DTG) is a guanidine derivative that has been detected in water [2, 24, 25] and examined in terms of its toxicity [26, 27].

The high polarity of PMOCs complicates their detection and analysis [28–31]. Moreover, reliable monitoring data are limited because of the lack of methods for the extraction and determination of PMOCs in aqueous media [1, 24]. Consequently, the development of methods that can accurately analyze PMOCs is urgently required [32]. To date, the analysis and quantitation of DPG remain difficult. For example, in a multi-layer solid-phase extraction (SPE) study, DPG recovery decreased from ~ 80% in tap water to 50% in effluent [17], and the liquid chromatography–high-resolution mass spectrometry (LC-HRMS) analysis of DPG was hindered by ion suppression due to the presence of Na^+^ and Cl^−^ [32]. Most existing studies on DPG analysis did not employ isotope-labeled DPG, and the reduced accuracy of the analyses was ascribed to a low recovery and the matrix effect. Hence, the use of isotope-labeled internal standards is recommended to achieve a highly accurate PMOC analysis [21].

In this study, we developed a novel analytical method based on LC–tandem mass spectrometry (LC–MS/MS) to quantify guanidine derivatives (including DPG and DTG) in surface water. Labeled DPG was used as a surrogate to account for the extraction loss and matrix effect. Other guanidine derivatives, i.e., 1-(*o*-tolyl)biguanide (TBG), cyanoguanidine (CG), 1-(4-cyanophenyl)guanidine (CPG), and *N*,*N'''*-1,6-hexanediylbis(*N'*-cyanoguanidine) (HCG), also exhibit PMOC characteristics; therefore, they were included in our study. Furthermore, we conducted a field survey on Western Japanese water sources, which provide drinking water to 12 million people [33], to determine seven guanidine derivatives using our method. This is the first guanidine-derivative PMOC survey, as well as the first survey of DPG in the Asian region.

## Materials and Methods

### Materials

Six guanidine derivatives (PMOC score = 3–5), namely DPG, DTG, TBG, CG, CPG, and HCG, were selected from the PMOC list of Arp et al. [6]. 1,2,3-Triphenylguanidine (TPG), which was not defined as a PMOC by Arp et al., was also used as a target analyte, as this compound is a vulcanization accelerator used in settings similar to those of DPG. Electronic Supplementary Material (ESM) Table S1 lists the target analytes and their selected properties. The values of p*K*a and log*D* (pH 7.0) were calculated using ChemAxon (https://chemaxon.com/products/calculators-and-predictors). Native standards were purchased from Sigma-Aldrich (St. Louis, MO, USA), Acros Organics (Geel, Belgium), Tokyo Chemical Industry (Tokyo, Japan), and BLDpharm (Shanghai, China). The surrogate standards, *N,N'*-diphenylguanidine-*d*_10_ (DPG-*d*_10_) and cyanoguanidine-^15^*N*_4_ (CG-^15^*N*_4_), were purchased from Toronto Research Chemicals (Toronto, ON, Canada). Methanol and acetonitrile for LC–MS were purchased from Kanto Chemical Co., Inc. (Tokyo, Japan). Ultrapure water for quadrupole time-of-flight mass spectrometry (QTOFMS) and formic acid for LC–MS were purchased from Fujifilm Wako Pure Chemical Corporation (Osaka, Japan). Ammonium formate solution (1 M) for high-performance LC was purchased from Nacalai Tesque, Inc. (Kyoto, Japan). Stock standard solutions (1 mg mL^−1^) were prepared in acetonitrile:water (1:1, v/v; HCG) or pure acetonitrile (other analytes) and stored at 4 °C (CG, CG-^15^*N*_4_, and HCG) or − 20 °C (other analytes).

Working solution A contained a mixture of the standard solutions, with the concentrations of DPG, DTG, TBG, TPG, CPG, and HCG being 500 ng mL^−1^ and that of CG being 5 μg mL^−1^; working solution B contained 20 ng mL^−1^ of DPG, DTG, TBG, TPG, CPG, and HCG, and 200 ng mL^−1^ of CG. These working solutions were used in the experiments described in the “Selection of optimal SPE cartridges”, “SPE clean-up with aqueous methanol”, and “Method validation and quality assurance/quality control (QA/QC)” sections.

### Development of the analytical method

#### Optimization of the LC column for target analyte analysis

The optimal LC column was selected among three hydrophilic interaction liquid chromatography (HILIC) columns (Waters ACQUITY UPLC BEH Amide, GL Sciences Inertsil® HILIC, MACHEREY-NAGEL NUCLEODUR HILIC), one mixed-mode liquid chromatography (MMLC) column (Thermo Acclaim™ Trinity P1), and one reversed-phase liquid chromatography (RPLC) column (Waters ACQUITY UPLC HSS T3), which have been used in previous PMOC studies [2, 17, 23, 25, 32]. Each column was examined using the same LC–MS/MS equipment, as described in the “Instrumental analysis” section. An appropriate solvent was selected for the mobile phase and the sample solvent based on previous studies conducted for each column [2, 17, 23, 25, 32]. The column dimensions and LC conditions for each column are listed in ESM Table S2.

Iterative measurements (*n* = 8) of the mixed standard solution at a signal-to-noise ratio of ~ 10 were conducted for each LC column, and the acquired data were used to estimate the instrument detection limits (IDLs) of the target analytes using the following equation [34, 35]:

$$\mathrm{IDL }\left(\mathrm{ng}\;\mathrm L^{-1}\right)={2t}_{1-\alpha,v}\sigma_0$$

where *σ*_0_ is the standard deviation of the iterative measurements and *t*_1-α,ν_ is the *t* value of the one-tailed test at *n* – 1 degrees of freedom and a significance level of 5%. For eight iterative measurements, the *t*_1-α,ν_ was estimated as 1.8946. The injection volumes (IVs) varied from 2 to 10 μL on each column; therefore, the IDL was expressed as the on-column injection amount in picograms to account for these differences as follows:

$$\mathrm{IDL}\left(\mathrm{pg}\right)=\mathrm{IDL}\left(\mathrm{ng}\;\mathrm L^{-1}\right)\times\mathrm{IV}(\mathrm{mL})$$

If a peak was detected in the blank for a target analyte, the IDL of the blank was calculated, and the higher IDL value between those of the standard and blank was adopted.

The retention factors (*k*′) of the target analytes were calculated for each column using the equation

$${k}^{\mathrm{^{\prime}}}=({t}_{\mathrm{r}}-{t}_{0})/{t}_{0}$$

where *t*_r_ is the analyte retention time and *t*_0_ is the column void time. Initially, *t*_0_ was calculated using the column volume and flow rate values. However, these values were higher than the observed *t*_r_, and the *k'* could not be calculated. Therefore, we estimated *t*_0_ values from the negative peak attributed to the difference in solvent between the sample and the mobile phase (BEH Amide and HSS T3 columns) or by analyzing an unretained standard (Inertsil® HILIC, NUCLEODUR HILIC, and Trinity P1 columns; for example, polycyclic aromatic hydrocarbon standards in *n*-nonane were analyzed using HILIC and mixed-mode columns).

#### Selection of optimal SPE cartridges

Six SPE cartridges—two mixed-mode (reversed-phase and ion-exchange) sorbents (Waters Oasis WCX Plus and Waters Oasis MCX Plus), two reversed-phase sorbents (Waters Oasis HLB Plus and Waters Sep-Pak PS2 Plus), one carbon sorbent (Supelco Supelclean™ ENVI-Carb™), and one activated carbon sorbent (Waters Sep-Pak AC2 Plus)—were examined. For cartridge evaluation, working solution A (0.05 mL) was added to 10 mL of ultrapure water (QTOFMS grade, Fujifilm Wako Pure Chemical Corporation), and recovery tests were then performed (*n* = 3). The surrogates were added to the reconstituted eluates as internal standards. ESM Table S3 lists the protocols used to evaluate each cartridge. The SPE cartridges, elution solvents, and experimental protocols were selected based on a previous study [2]. With the exception of CG, good results (90–100% recoveries) were obtained for all target analytes (ESM Table S3, WCX). CG was only slightly retained on AC2 (ESM Table S3, AC2 protocol 1) under the initially investigated protocols. Therefore, two additional solvents were examined as AC2 eluents (ESM Table S3, AC2 protocols 2 and 3) based on previous studies [36, 37].

#### Blank tests for SPE cartridges and ultrapure water of various grades

Blank tests were performed for various grades of ultrapure water and the optimal SPE cartridges (selected based on the results in the “Selection of optimal SPE cartridges” section), i.e., the WCX and AC2 SPE cartridges (*n* = 1). Six grades of ultrapure water were examined, namely LC–MS, QTOFMS, and perfluorooctane sulfonate (PFOS)-perfluorooctanoic acid (PFOA) grade water obtained from Fujifilm Wako Pure Chemical Corporation (Osaka, Japan); ultrapure grade and LC–MS grade water obtained from Kanto Chemical Co., Inc. (Tokyo, Japan); and water prepared using a PURELAB Flex-3 (VWS Ltd., UK) purification system in our laboratory. Ultrapure water samples (100 mL) were spiked with surrogates prior to the extraction and extracted in accordance with the optimized sample preparation method described in the “Final optimized sample preparation and analysis procedures” section. The blank SPE cartridges were connected in series, loaded with 10 mL of the wash solvent (ultrapure water for QTOFMS, Fujifilm Wako Pure Chemical Corporation), and eluted using the series of solvents described in the “Final optimized sample preparation and analysis procedures” section. The eluates were spiked with the surrogates and examined. For ease of comparison, the results were calculated based on the assumption that 100 mL of water was used in the experiment.

#### SPE clean-up with aqueous methanol

During the SPE cartridge optimization (“Selection of optimal SPE cartridges” section), ultrapure water was the most frequently used wash solvent prior to elution (ESM Table S3). If the target analytes could be retained when aqueous methanol was used as a wash solvent, certain interfering compounds could potentially be removed. Therefore, we examined the effects of 10–60 vol% aqueous methanol solutions as the wash solvent. Working solution A (0.05 mL) was added to 10 mL of ultrapure water (QTOFMS grade, Fujifilm Wako Pure Chemical Corporation), and the mixture was loaded onto the WCX and AC2 SPE cartridges. The cartridges were washed with 20 mL of 10–60 vol% aqueous methanol (*n* = 3) and were then eluted using the solvent series described in the “Final optimized sample preparation and analysis procedures” section. Ultrapure water (QTOFMS grade, Fujifilm Wako Pure Chemical Corporation) was used as the blank for the wash step (*n* = 3). Surrogates were added to the reconstituted eluates as internal standards, and target analyte recoveries were subsequently examined. The target analyte recoveries in the wash blank tests were defined as 100% (ultrapure water was used as the wash solvent), and the recoveries for aqueous methanol were calculated in proportion to those of the blank tests.

Recovery (%) = Target analyte concentration for aqueous methanol (ng L^−1^)/Target analyte concentration in the blank tests (ng L^−1^) × 100.

#### Instrumental analysis

Guanidine derivatives were quantified using an ACQUITY UPLC system coupled with a Xevo-TQ triple quadrupole mass spectrometer (Waters Corp., MA, USA). Analyte quantitation was performed by positive-ion electrospray ionization mass spectrometry in multiple reaction monitoring (MRM) mode; the MRM transitions of the target analytes and the corresponding surrogate standard for each target analyte are listed in ESM Table S4.

### Method validation and quality assurance/quality control (QA/QC)

The analytical precision of our method was tested using field samples. Ten replicate analyses of a river water sample (Yodo River) were conducted: three were non-spiked samples, and each of the remaining seven was spiked with standards. For the recovery studies, the river water samples were spiked with working solution B (0.05 mL). The spiked amount of CG was tenfold greater than those of the other target analytes because CG was detected in non-spiked river water at a level of 167 ng L^−1^. The sampling procedure and sample descriptions used for QA/QC are described in the “Sampling” section and ESM Table S6, respectively. Our sample preparation method and the analytical conditions used for QA/QC are described in the “Final optimized sample preparation and analysis procedures” section and ESM Tables S4 and S5.

### Sampling

Lake water (three samples; LW-1–3), river water (four samples; RW-1–4), sewage effluent (five samples; SE-1–5), and tap water (two samples; TW-1–2) samples obtained from Western Japan in July 2021 were investigated. Two of the sampling locations represent drinking water sources in the Kansai district. The sampling sites of LW-1–3 were located in the center of the lake, whereas those of RW-1–4 were located in an urban area. Therefore, the sampling sites of RW-1–4 were considered to be more frequently affected by road drainage compared with those of LW-1–3. Detailed sample descriptions are provided in ESM Table S6. Information on the water height and rainfall amounts at the sampling sites (lake and river) prior to sampling is provided in ESM Fig. S1.

Initially, lake water was sampled using a Van Dorn sampler with a rubber component (RIGO, Japan). However, the rubber component contained DPG, which interfered with the quantitation of DPG in the water samples. Hence, a RIGO-B transparent water bottle (acrylic resin/polycarbonate/polyvinyl chloride) was used for sampling to avoid DPG contamination. This procedure is described in detail in the ESM (examination of water sampling methods). River water was sampled using a stainless steel bucket. Tap water was sampled directly into a glass sample bottle. Each water sample was immediately treated with sodium thiosulfate (~ 0.05 g) for residual chlorine removal, which was particularly important for tap water.

## Results and discussion

### Analytical method development for guanidine derivative quantitation

#### Selection of the LC column

Table 1 lists the IDLs of the target analytes determined using five LC columns, in which the highest values were obtained for the HSS T3 column. The HSS T3 column has a high-strength silica-based C_18_ as the stationary phase, which enables the retention of highly polar compounds [38]. The IDLs of DPG, DTG, and TPG obtained for the HSS T3 column were 2–3 orders of magnitude higher than those obtained for the other columns. In the case of DPG, DTG, and TPG, the IDLs of the blanks were higher than those of the standards, and the IDLs of the blank were adopted for the HSS T3 column. The higher IDLs of these compounds could be attributed to the large fluctuation in the blank peaks of these compounds on this column. Therefore, the HSS T3 column was rejected. The IDL of CG was the highest among the target analytes for the three HILIC and the Trinity P1 columns, where the IDL of CG for the Trinity P1 column was one order of magnitude higher than those obtained for the HILIC columns. The Trinity P1 column has a nanopolymer hybrid silica-based mixed-mode stationary phase (reversed-phase/anion exchange/cation exchange). Schulze et al. [2] reported that the IDL of CG obtained for the Trinity P1 column was two orders of magnitude higher than those obtained for the BEH Amide and HSS T3 columns, which is consistent with our experimental results. Therefore, the Trinity P1 column was also rejected, and the three HILIC columns were selected for further investigation. The BEH Amide, Inertsil HILIC, and NUCLEODUR HILIC columns contain high polarity amide groups [39], diol (dihydroxypropyl) groups, and ammonium-sulfonic acid betaine zwitterions, respectively. The IDLs of the target analytes for the three HILIC columns were comparable, indicating that the IDL values do not depend on the stationary phase ligands of the columns.

**Table 1:** Instrument detection limits (pg) of target analytes determined for five LC columns under the conditions listed in ESM Table S2

Substance	HILIC			MMLC	RPLC
	BEH Amide	Inertsil	NUCLEODUR	Trinity P1	HSS T3
DPG	0.051	0.15	0.10	0.064	10
DTG	0.14	0.062	0.013	0.060	8.2
TPG	0.35	0.41	0.020	0.098	13
TBG	0.52	5.1	0.061	0.20	3.2
CG	62	91	69	280	37
CPG	0.13	0.37	0.30	0.79	5.7
HCG	1.1	3.8	0.95	32	1.1

ESM Fig. S3 presents the calculated *k*′ values, showing that in all cases except CG, the values for the HILIC columns were lower than those for the MMLC and RPLC columns, whereas the reverse was true for CG. Notably, the *k*′ values obtained for the NUCLEODUR column (*k*′: 0.20–2.15) were comparable or up to six times higher than those obtained for the other two HILIC columns. Therefore, the NUCLEODUR HILIC column was selected for further use.

#### Effects of SPE sorbent on analyte recovery

**Fig. 1:**
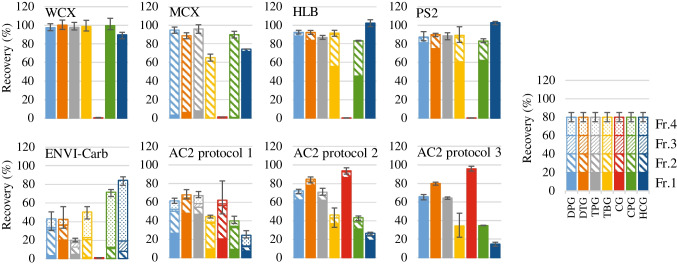
Recoveries of target analytes determined for the six SPE cartridges (*n* = 3) using the protocols listed in ESM Table S3. The error bars indicate the maximum and minimum recoveries of triplicate analyses. The bar graph design indicates the elution fractions of each eluent

Figure 1 presents the target analyte recoveries obtained for the six SPE cartridges, revealing that values > 80% were observed for all the target analytes except CG when the weak-cation-exchange sorbent (WCX) and two reversed-phase sorbents (HLB and PS2) were used. The sorbent in the HLB cartridges is a lipophilic divinylbenzene–hydrophilic *N*-vinylpyrrolidone copolymer and that of PS2 is a styrene–divinylbenzene copolymer [40]. These sorbents exhibit better retention toward polar compounds than do octadecyl-silica (ODS) sorbents. They can also more readily elute basic compounds compared to ODS because they have no residual silanol groups, whereas ODS has residual silanol groups that undergo undesirable interactions with basic compounds. Consequently, HLB and PS2 exhibited relatively good recoveries for the target analytes except CG, despite the presence of reversed-phase sorbents. The divinylbenzene–*N*-vinylpyrrolidone copolymer is used in HLB, WCX, and MCX cartridges. In addition, WCX and MCX contain substituted ion-exchange sorbents comprising carboxylic and sulfonic acids, and hence retain strongly basic compounds of p*K*_a_ > 10 and p*K*_a_ = 2–10, respectively [41]. As shown in ESM Table S1, the p*K*_a_ values of the target analytes, except that of CG, are in the range 5.26–10.22. However, MCX exhibited lower recoveries of TBG and HCG (65 and 74%, respectively) compared to those of WCX. This is attributed to the adsorption strength of the sulfonic acid groups in MCX, which do not easily release compounds with higher p*K*_a_ values, such as TBG (p*K*_a_ 10.22) (ESM Table S1). Consequently, WCX demonstrated the highest recoveries (90–100%) among all the SPE cartridges for all tested analytes except CG. The WCX pass-through and wash fractions after sample loading contained 82% of the loaded CG (data not shown). Therefore, WCX is a suitable sorbent for all the tested analytes except CG.

The recovery of CG for all SPE cartridges except AC2 (62%) was ≤ 1% (Fig. 1, AC2 protocol 1). Therefore, two additional solvents were examined as AC2 eluents, acetonitrile:methanol (3:2, v/v) and acetonitrile:water (9:1, v/v) (ESM Table S3, AC2 protocols 2 and 3). Notably, CG was retained by AC2 with a recovery of 96% when the latter eluent was used (Fig. 1, AC2 protocol 3). AC2 has been previously used to enrich very polar compounds in water, e.g., 1,4-dioxane [42, 43] and *N*-nitrosodimethylamine [44]. As a polar compound, CG is also strongly retained by AC2 when an appropriate eluent is used [36, 37]. When acetonitrile:methanol (3:2) was used as the eluent, CG was retained by AC2 with a recovery of 94% (Fig. 1, AC2 protocol 2). In terms of eluent volume, 10 mL of acetonitrile:water (9:1) was required for complete CG elution, whereas the required volume of acetonitrile:methanol (3:2) was 30 mL. Therefore, acetonitrile:water (9:1) was selected as the AC2 eluent. WCX and AC2 cartridges were connected in series to retain the target analytes.

#### Blank test results of SPE cartridges and various types of ultrapure water

ESM Fig. S4 presents the blank test results of the SPE cartridges and various ultrapure water samples. In the SPE cartridge blanks, only DPG was detected at a level of 0.25 ng L^−1^. In the six ultrapure water samples, DPG and CG were detected at levels of 0.39–0.69 and 80–150 ng L^−1^, respectively. Some studies have reported the contamination of blanks with DPG [30], or DPG and DTG [2, 24]. In our laboratory, the target analytes were not detected in the instrumental blank (data not shown). However, DPG was detected in the SPE cartridge blank and in all the ultrapure water samples at very low concentrations; the source of this contamination is unknown. The best strategy to address this problem is to carefully monitor the SPE cartridge and ultrapure water blanks for each analytical batch and compare the sample and blank concentrations to prevent false positive results.

Although CG was not detected in the SPE cartridge blank, it was detected in two ultrapure water samples at sub-ppb levels. The PURELAB Flex-3 reverse osmosis module was used to generate ultrapure water from municipal tap water. As described in the “Analysis of guanidine derivatives in water samples” section, CG was detected at a level of ~ 100 ng L^−1^ in tap water sampled in Western Japan and could not be removed by the reverse osmosis module of the water purification system, possibly because of the low molecular weight of CG (84.1 Da). Thus, we avoided the use of CG-contaminated ultrapure water in our study.

Based on these results, we selected the ultrapure water with the lowest blank level and used QTOFMS grade ultrapure water in our experiments. The presence of DPG and CG in the blank tests suggests that the levels of these contaminants should be decreased to enable their accurate monitoring.

#### SPE clean-up using aqueous methanol

**Fig. 2:**
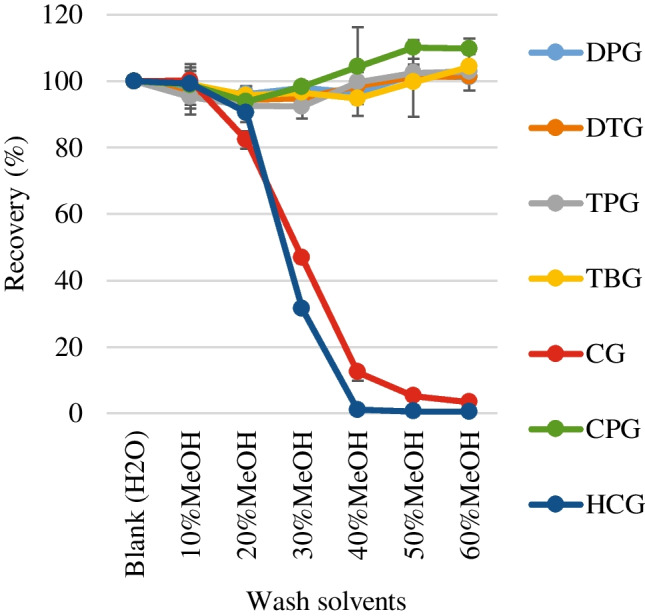
Recoveries of target analytes obtained for different wash steps using aqueous methanol as the wash solvent (*n* = 3). Horizontal axis labels indicate the solvents used to wash the SPE cartridges after sample loading. Ultrapure water was used as the blank in the wash step. The error bars indicate the maximum and minimum recoveries of triplicate analyses

Figure 2 presents the recoveries of the target analytes when aqueous methanol was used as the wash solvent during wash step, with the result obtained for ultrapure water (blank) as the wash solvent shown as a reference.

Except for CG and HCG, the target analytes were retained when the SPE cartridges were washed with 10–60 vol% aqueous methanol. In contrast, the recoveries of CG and HCG began to decrease at methanol contents of 20 vol%, sharply decreased with an increase in methanol content up to 40 vol%, and then gradually decreased and reached saturation at a methanol content of 60 vol%. Target analytes except CG were retained by WCX, which comprises weak-cation-exchange and reversed-phase sorbents. As shown in ESM Table S1, the p*K*_a_ values of the target analytes except CG are 5.26–10.22. We deduced that DPG, DTG, TPG, TBG, and CPG, which have p*K*_a_ values in the range 8.51–10.22, were retained by the ion-exchange component of WCX because these compounds did not elute even when 60 vol% methanol was used. In contrast, HCG (p*K*_a_ 5.26) may have been retained by the reversed-phase component of WCX because its elution rate increased with an increase in the methanol content. We observed that methanol content should be lower than 10% to retain CG and HCG. However, washing the SPE sorbents with less than 10 vol% aqueous methanol will result in poor purification. Based on these results, we rejected using 10–60 vol% aqueous methanol for the wash step.

### Final optimized sample preparation and analysis procedures

After comprehensive evaluation of the sample preparation and analysis parameters, we determined the optimal method for the extraction and detection of guanidine derivatives in water. Prior to extraction, a 100-mL water sample was spiked with 5 and 50 ng of DPG-*d*_10_ and CG-^15^*N*_4_, respectively, as surrogates for the target analytes (ESM Table S4). The spiked sample was loaded onto a mixed-mode weak-cation-exchange (Oasis WCX Plus, Waters) SPE cartridge connected in series to an activated carbon (Sep-Pak AC2 Plus, Waters) SPE cartridge at a flow rate of ~ 5 mL min^−1^. The WCX cartridge was pre-conditioned with 5 mL of aqueous ammonia:methanol (5:95, v/v) and 5 mL of ultrapure water (QTOFMS grade, Fujifilm Wako Pure Chemical Corporation), whereas the AC2 cartridge was treated with 10 mL of acetonitrile and 10 mL of ultrapure water. The former cartridge was located above the latter. After sample loading, the cartridges were washed with 10 mL of ultrapure water and dried. The analytes were separately eluted from the two cartridges using different solvents. The basic compounds, DPG, DTG, TPG, TBG, CPG, and HCG, were eluted from WCX with 5 mL of 2 vol% formic acid in methanol by back flushing. CG was eluted from AC2 with 10 mL of acetonitrile:ultrapure water (9:1) by back flushing under gravity. Each extract was separately evaporated to dryness under N_2_ and reconstituted in 500 μL of acetonitrile:ultrapure water (95:5) containing 5 mM ammonium formate. Each reconstituted extract was treated in an ultrasonic bath for 5 min and filtered through a 0.20-μm hydrophilic PTFE syringe filter (DISMIC 13HP020AN; Advantec Co., Ltd., Japan). Two eluates of WCX and AC2 cartridges were obtained for every sample and were separately analyzed.

The separation of target analytes was achieved using the optimal HILIC column (MACHEREY-NAGEL NUCLEODUR HILIC) and an acetonitrile–water gradient buffered with ammonium formate at pH 3; the gradient conditions are listed in ESM Table S5. Quantitation was performed using an isotope dilution method with DPG-*d*_10_ and CG-^15^*N*_4_ as internal standards to account for analyte losses during extraction. The detailed mass spectrometric parameters are listed in ESM Table S5. The column was equilibrated (using the gradient B conditions detailed in ESM Table S5) for a suitable time to prevent the carryover of the target analytes.

### Method validation and QA/QC results obtained using field samples

The linearities of the calibration curves (*r* > 0.995) were confirmed in the ranges 0.05–250 ng mL^−1^ for DPG, DTG, TPG, and TBG; 0.5–250 ng mL^−1^ for CPG and HCG; and 5–2500 ng mL^−1^ for CG. The method detection limits (MDLs) of the target analytes were calculated from the lowest concentrations (with the signal-to-noise ratios of at least 10) of the calibration curves and the sample preparation volumes [15]. The lowest standard concentration of 0.05 ng mL^−1^ for DPG, DTG, TPG, and TBG corresponded to a concentration of 0.25 ng L^−1^ in a water sample; 0.5 ng mL^−1^ for CPG and HCG corresponded to 2.5 ng L^−1^; and 5 ng mL^−1^ for CG corresponded to 25 ng L^−1^. To validate our method in real samples, we used water samples from the Yodo River in Osaka Prefecture, Japan. This river is a drinking water source for approximately 12 million people in the Kansai district of Japan [33]. Its watershed is highly urbanized and industrialized; therefore, it contains various environmental contaminants, including polar and non-polar compounds such as PFOA and hexabromocyclododecane, respectively [45, 46]. Therefore, we examined our analytical method using the Yodo River samples as the real matrix.

Table 2 lists the recoveries of the target analytes. Chromatograms of target analytes in non-spiked and standard-spiked river water are shown in ESM Fig. S5. The detected concentrations of DPG and CG were 13.4 and 167 ng L^−1^, respectively, whereas those for DTG and TBG were below 1 ng L^−1^ in non-spiked river water. The recoveries of the spiked standards, DPG, DTG, TPG, TBG, CG, CPG, and HCG, were 72.7, 100.8, 94.5, 107.9, 107.7, 89.8, and 137.0%, and the corresponding coefficients of variation were 2.2, 3.4, 5.8, 4.0, 3.9, 4.8, and 2.1%, respectively. HCG recovery was > 130%, which was the highest among the analytes. The adsorption behavior of HCG is different from that of DPG-*d*_10_, which was used as the internal standard to quantify HCG. HCG undergoes reversed-phase adsorption, whereas DPG, DTG, TPG, TBG, CPG, and DPG-*d*_10_ proceed via ion-exchange adsorption on the WCX sorbents, as elucidated in the “SPE clean-up using aqueous methanol” section. Therefore, the quantification of HCG exhibited limitations on account of the matrix effect when the procedure for DPG-*d*_10_ was used. The DPG recovery was lower than that of the other analytes because the spiked concentration was approximately the same as that in the non-spiked river water. In contrast, the recovery of CG was 107.7% despite also being present in the non-spiked river water. Therefore, CG, which is retained by AC2, could have been less influenced by the matrix effect than were the other analytes retained by WCX because AC2 was located below WCX, which would adsorb most of the organic matter in the matrix before the sample reached AC2. These results confirmed the robustness and accuracy of the developed method and revealed its suitability for the quantitation of guanidine derivatives in aquatic environments.

**Table 2:** Recoveries of target analytes

Analyte	Concentration in sample (ng L^−1^)^a^	Amount added (ng L^−1^)	Concentration in spiked sample (ng L^−1^)^b^	Recovery (%)	Standard deviation (ng L^−1^)	Coefficient of variation (%)
DPG	13.4	10	20.7	72.7	0.45	2.2
DTG	0.52	10	10.6	100.8	0.36	3.4
TPG	< 0.25	10	9.45	94.5	0.55	5.8
TBG	0.35	10	11.1	107.9	0.44	4.0
CG	167	100	275	107.7	10.8	3.9
CPG	< 2.5	10	8.98	89.8	0.43	4.8
HCG	< 2.5	10	13.7	137.0	0.29	2.1

^a^Concentrations are averages of three non-spiked samples. ^b^Concentrations are averages of seven spiked samples.

### Comparison between our method and previously reported methods

Previous studies have investigated some of the same target analytes as those in this study. Therefore, we compared our method with the previously reported methods (Table 3). DPG, DTG, and CG have been analyzed in previous studies as a part of PMOCs [2, 17, 20, 21, 30]. Moreover, DPG is a water contaminant that originates from tires [16, 19]. To the best of our knowledge, an analytical quantitation method for guanidine derivative PMOCs has not been developed thus far. We developed a quantitation method for TPG, TBG, CPG, and HCG in water and reported TBG and HCG concentrations for the first time, as mentioned in the “Analysis of guanidine derivatives in water samples” section. Furthermore, even though DPG and CG have been extensively analyzed in previous studies, our method established a highly accurate quantification using the surrogate compounds DPG-*d*_10_ and CG-^15^*N*_4_. DPG-*d*_10_ has been used in one study [20], whereas CG-^15^*N*_4_ has not been used to date. We observed 73–137% recoveries from seven replicate analyses in our recovery study using DPG-*d*_10_ and CG-^15^*N*_4_, confirming its analytical accuracy and precision. Our number of recovery studies (seven replicate analyses) was greater than those of the other studies (three or four replicate analyses), which indicates that our data is more reliable. The quantitation limits of the target analytes were comparable to those of previous studies that used mass spectrometry [2, 15, 19–21].

**Table 3:** Comparison between our method and previously reported methods

	Target analytes	Number of target analytes	Analysis devices	Surrogate compounds for target analytes	Quantitation limits	Quality assurance/quality control methods for recovery studies	Recoveries
Our method	Guanidine derivative PMOCs	7	LC–MS/MS	DPG-*d*_10_ and CG-^15^*N*_4_	0.25 ng L^−1^ (DPG, DTG, TPG, and TBG) 25 ng L^−1^ (CG) 2.5 ng L^−1^ (CPG and HCG)	Seven replicate analyses of recovery studies using river water	73–137% (CV: 2.1–5.8%)
Johannessen et al. [15]	Tire wear compounds including DPG	4	LC-HRMS	‒	9.8 ng L^−1^ (DPG)	Three replicate analyses of recovery studies using tap water	DPG: 93 ± 8%
Köke et al. [17]	Polar organic compounds including DPG	26	LC–MS/MS	‒	Unknown	Three replicate analyses of recovery studies using tap water, surface water, and wastewater effluent	DPG: > 80% (tap water), > 60% (surface water), ~ 50% (wastewater effluent)
Neuwald et al. [30]	PMOCs including DPG, DTG, CG, and HCG	1310 (Suspect screening)	LC-HRMS and SFC-HRMS	‒	Unknown	Not performed (semi-quantitative concentration)	‒
Peter et al. [19]	Stormwater-derived compounds including DPG	35	LC–MS/MS (for quantification)	17 surrogate compounds, but not DPG-*d*_10_	0.74–4.3 ng L^−1^ (DPG)^a^	Not performed	‒
Scheurer et al. [20]	PMOCs including DPG and DTG	25	LC–MS/MS	DPG-*d*_10_	8.7 ng L^−1^ (DPG) 5.0 ng L^−1^ (DTG)	Three replicate analyses of recovery studies using surface water	DPG: 92%, DTG: 94%
Schulze et al. [2]	PMOCs including DPG, DTG, and CG	57	LC–MS/MS and SFC-HRMS	‒	0.5 ng L^−1^ (DPG), 0.28 ng L^−1^ (DTG), 20 ng L^−1^ (CG)^b^	Not performed (semi-quantitative concentration estimation)	‒
Schulze et al. [21]	PMOCs including DPG and CG	17	SFC-HRMS	‒	71 ng L^−1^ (DPG) 61 ng L^−1^ (CG)	Four replicate analyses of recovery studies using river water	DPG: 84% (CG: no data)
Smith and Schallenberg [47]	CG	1^c^	Spectrophotometer	‒	25 μg L^−1^	Not performed	‒

^a^The quantification limits were recalculated on each analysis date. ^b^Plural methods and their quantitation limits were adopted in this study; therefore, the lowest value among the quantitation limits was chosen for DPG, DTG, and CG. ^c^Inorganic nitrogen compounds were not considered as target analytes in the water samples.

Although the number of target analytes in our study (seven analytes) is smaller than those used in most of the other studies, our method exclusively focused on selected PMOCs, i.e., the guanidine derivatives. Further research is necessary to expand the scope of our method to other PMOC target analytes. Moreover, only DPG and CG were commercially available as isotope-labeled compounds at the time of the study; therefore, to increase the analytical accuracy and precision of the quantitation of DTG, TBG, and HCG, isotope-labeled counterparts of these compounds are necessary.

### Analysis of guanidine derivatives in water samples

**Fig. 3:**
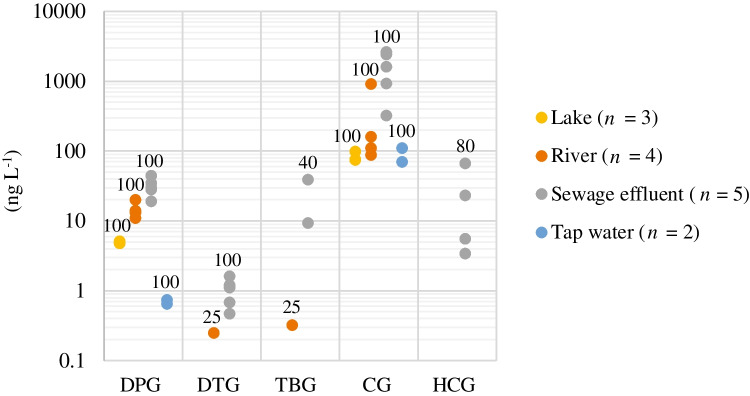
Concentration ranges of target analytes determined for water sampled in July 2021 in Western Japan. TPG and CPG were not detected in all samples. The values added above the data points in the plot indicate the detection frequency percentage

Figure 3 presents the concentration ranges of the target analytes detected in the water samples. Among the seven guanidine derivatives, five (DPG, DTG, TBG, CG, and HCG) were detected in at least three samples, whereas two (TPG and CPG) were not detected in any sample. DTG, TBG, and HCG were primarily detected in the sewage effluent. DPG and CG were detected in all the samples including tap water at levels of up to 44 and 2600 ng L^−1^, respectively. This is the first study to report the presence of DPG in the surface water of Japan. Lake water was also analyzed to estimate the background levels of the target analytes, and only DPG and CG were detected. Thus, our results indicate the ubiquitous contamination of aquatic environments with these compounds.

Schulze et al. [2] performed the semi-quantitative analysis of PMOCs, including DPG and CG, in 14 water samples, including river water, groundwater, and riverbank filtrate, and reported that DPG was detected in all 14 samples at levels of up to 100 ng L^−1^, whereas CG was detected in all seven river water samples and one ground water sample at levels of up to > 3000 ng L^−1^. The results obtained in our study for DPG and CG were comparable with those of Schulze et al. CG was detected at levels of up to 946 µg L^−1^ in drainage water in New Zealand in another study [47]. Neuwald et al. [30] detected CG in 11 investigated surface water samples and described CG as a novel or scarcely investigated water contaminant. CG was also detected in tap water in our investigation. CG cannot be removed by conventional or advanced (e.g., those using ozone and activated carbon) water treatment processes [5]. In this study, CG was detected in all the analyzed samples, which indicates its ubiquitous presence in surface water and explains its presence in tap water.

Zahn et al. [23] detected DPG in all their investigated samples, with similar results reported by Johannessen et al. [15, 16], Scheurer et al. [20], and Tian et al. [22]. DPG was also detected in roadway runoff [11, 14–16, 19]. However, Johannessen et al. [16] noted that road runoff may not be the only source of DPG because of its omnipresence in surface waters. It had not rained for four days prior to our lake sampling, as shown in ESM Fig. S1. However, it rained 3 days prior to river sampling, and a corresponding increase in the water height in locations RW-1–4 was observed, which reverted to a stable condition on the sampling day. Therefore, we assumed that the rain did not affect our survey results. Nevertheless, pre- and post-rainfall studies should be conducted in the future because the presence or absence of rainfall affects the concentration of DPG. Furthermore, the occurrence and spatial distribution of the target analytes in water were beyond the scope of this study. Thus, further studies are required to determine the distribution, fate, and emission sources of DPG in water and its adverse effects on aquatic biota.

According to Sieira et al. [13], DPG and DTG rapidly react with chlorine during water purification. In our preliminary experiments, the recovery of DPG-*d*_10_ in tap water was only 1%, which was ascribed to the chlorination of DPG-* d*_10_ by the residual chlorine (data not shown). Therefore, in subsequent experiments, sodium thiosulfate was added to all the samples to quench the residual chlorine and thus prevent further chlorination. Sieira et al. [13] also reported that certain chlorinated DPG and DTG derivatives may be more toxic than their unchlorinated counterparts. Although we did not detect DTG in tap water, the presence of chlorinated DTG and DPG cannot be excluded. Therefore, further studies should focus on identifying chlorinated DPG and DTG in tap water.

## Conclusion

In this study, we developed a novel analytical method that combines solid-phase extraction and LC–MS/MS for the accurate quantitation of seven guanidine derivatives in aquatic environments. Five LC columns (three HILIC columns, one MMLC, and one RPLC column) were examined, and the NUCLEODUR HILIC column was the most suitable owing to its favorable IDL and *k'*. A weak-cation-exchange sorbent (Oasis WCX Plus, Waters) was used to retain all the target analytes except for CG, which was retained by activated carbon (Sep-Pak AC2 Plus, Waters). DPG and CG were detected in ultrapure water blanks at levels of up to 0.69 and 150 ng L^−1^, respectively. The analytical precision of our method was assessed using seven replicate analyses of standard-spiked river water, with the corresponding analyte recoveries determined as 73–137% (coefficient of variation = 2.1–5.8%). The method was applied to the detection of guanidine derivatives in lake water, river water, sewage effluent, and tap water sampled in July 2021 in Western Japan. DPG and CG were detected in all samples including tap water at levels of up to 44 and 2600 ng L^−1^, respectively. This is the first reported detection of DPG in the surface water of Japan, and the first report in the literature of TBG and HCG detection in water. Our results indicate that aquatic environments are ubiquitously contaminated with the aforementioned compounds and suggest that further studies are necessary to determine the distribution, fate, and emission source of these compounds and to identify chlorinated DPG and DTG in tap water.

## Supplementary information

Below is the link to the electronic supplementary material.

Supplementary file1 (PDF 883 KB)

## Data Availability

The datasets generated during and/or analyzed during the current study are available from the corresponding author on reasonable request.

## Abbreviations

CG: Cyanoguanidine
HRMS: High-resolution mass spectrometry
HILIC: Hydrophilic interaction liquid chromatography
IV: Injection volume
IDL: Instrument detection limit
LC–MS/MS: Liquid chromatography-tandem mass spectrometry
MDL: Method detection limit
MMLC: Mixed-mode liquid chromatography
MRM: Multiple reaction monitoring
HCG: *N,N′′′*-1,6-Hexanediylbis(*N′*-cyanoguanidine)
ODS: Octadecyl-silica
PFOS: Perfluorooctane sulfonate
PFOA: Perfluorooctanoic acid
PMT: Persistent, mobile, and toxic
PMOC: Persistent and mobile organic compound
QTOFMS: Quadrupole time-of-flight mass spectrometry
QA/QC: Quality assurance/quality control
RPLC: Reversed-phase liquid chromatography
SCHEER: Scientific Committee on Health, Environmental and Emerging Risks
SFC: Supercritical fluid chromatography
SPE: Solid-phase extraction
TBG: 1-(*o*-Tolyl)biguanide
TPG: 1,2,3-Triphenylguanidine
DTG: 1,3-Di-*o*-tolylguanidine
DPG: 1,3-Diphenylguanidine
CPG: 1-(4-Cyanophenyl)guanidine
