# Supplementary material for: Quantitation of guanidine derivatives as representative persistent and mobile organic compounds in water: method development
Source: Anal Bioanal Chem. 2023 Feb 28;415(10):1953–65. doi: 10.1007/s00216-023-04613-x (PMC10050033; doi:10.1007/s00216-023-04613-x)
Supplement: Supplementary file 1 — Supplementary file1 (PDF 883 KB) [file 216_2023_4613_MOESM1_ESM.pdf]

Analytical and Bioanalytical Chemistry

Electronic Supplementary Material

Quantitation of guanidine derivatives as representative persistent and mobile organic compounds in water: Method development

Makiko Ichihara[a,*], Daichi Asakawa[a], Atsushi Yamamoto[b], and Miki Sudo[c]

[a]Osaka City Research Center of Environmental Science, 1-3-3 Nakamichi, Higashinari-ku, Osaka 537-0025, Japan

[b]Faculty of Environmental Studies, Tottori University of Environmental Studies, 1-1-1 Wakabadai-kita, Tottori, Tottori 689-1111, Japan

[c]Department of Biological Resources Management, School of Environmental Science, The University of Shiga Prefecture, 2500 Hassaka-cho, Hikone, Shiga 522-8533, Japan

***Corresponding author.** Tel.: +81-6-6972-9024, fax: +81-6-6972-9666, e-mail:mak-ichihara@city.osaka.lg.jp, ORCID: 0000-0002-5758-7741

Table of contents

**Table S1**	List of target guanidine derivatives	S1
**Table S2**	Conditions used to assess the tested LC columns	S2
**Table S3**	Experimental conditions used to evaluate the six SPE cartridges	S4
**Table S4**	Multiple reaction monitoring (MRM) transitions of target analytes	S5
**Table S5**	LC-MS/MS measurement conditions	S6
**Table S6**	Sample description	S7
**Fig. S1**	Rainfall amounts and water height at each sampling site (lake and river) prior to sampling	S9
**Examination of water sampling methods**		S10
**Fig. S2**	DPG concentrations determined in lake surface water sampled using different devices	S11
**Fig. S3**	Retention factors (*k*′) of target analytes obtained for the five examined LC columns	S12
**Fig. S4**	Blank test results of SPE cartridges and various ultrapure water samples	S13
**Fig. S5**	Chromatograms of target analytes in the method validation	S14

Table S1 List of target guanidine derivatives

Substance	Abbreviation	Formula	CAS No.	pK_a[a]	log*D* at pH 7.0[a]	Structure	Supplier	Purity
1,3-Diphenylguanidine	DPG	$C_{13}H_{13}N_3$	102-06-7	9.38	1.03	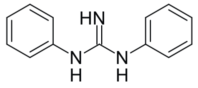	Sigma-Aldrich	97%
1,3-Di-*o*-tolylguanidine	DTG	$C_{15}H_{17}N_3$	97-39-2	9.43	2.03	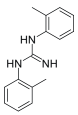	Sigma-Aldrich	99%
1,2,3-Triphenylguanidine	TPG	$C_{19}H_{17}N_3$	101-01-9	8.51	3.70	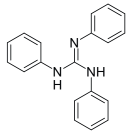	Sigma-Aldrich	–
1-(*o*-Tolyl)biguanide	TBG	$C_9H_{13}N_5$	93-69-6	10.22	−2.70	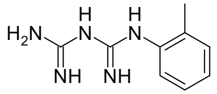	Sigma-Aldrich	98%
Cyanoguanidine	CG	$C_2H_4N_4$	461-58-5	5.15	−1.03	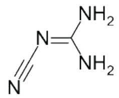	Acros Organics	99.5%
1-(4-Cyanophenyl)guanidine	CPG	$C_8H_8N_4$	5637-42-3	10.09	−1.53	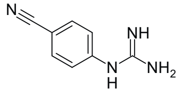	Tokyo Chemical Industry	>99.0%
N,N'''-1,6-Hexanediylbis(*N'*-cyanoguanidine)	HCG	$C_{10}H_{18}N_8$	15894-70-9	5.26	−0.12	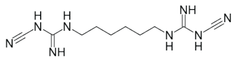	BLDpharm	98%

[a]pK_a and log*D* (pH 7.0) of the target analytes were calculated using ChemAxon (https://chemaxon.com/products/calculators-and-predictors).

Table S2 Conditions used to assess the tested LC columns

Chromatographic mode	Conditions
HILIC	*Column:* Waters ACQUITY UPLC BEH Amide (2.1 mm × 100 mm, 1.7 μm)
	Solvent A: acetonitrile:water (5:95) containing ammonium formate (5 mM), pH 3
	Solvent B: acetonitrile:water (95:5) containing ammonium formate (5 mM), pH 3
	Gradient B (%): 0–3 min, 100%; 3.1–9 min, 50%; 9.1–15 min, 100%
	Flow rate (mL min^{-1}): 0.2
	Column temp. (°C): 30
	Injection volume (μL): 2
	Sample solvent: acetonitrile:water (95:5)
HILIC	*Column:* GL Sciences Inertsil® HILIC (2.1 mm × 150 mm, 3 μm)
	Solvent A: acetonitrile:water (5:95) containing ammonium formate (5 mM), pH 3
	Solvent B: acetonitrile:water (95:5) containing ammonium formate (5 mM), pH 3
	Gradient B (%): 0–4 min, 100%; 4.1–10 min, 60%; 10.1–15 min, 100%
	Flow rate (mL min^{-1}): 0.2 (10.1–13 min; 0.4)
	Column temp. (°C): 30
	Injection volume (μL): 2
	Sample solvent: acetonitrile:water (95:5)
HILIC	*Column:* MACHEREY-NAGEL NUCLEODUR HILIC (2.1 mm × 150 mm, 3 μm)
	Solvent A: acetonitrile:water (5:95) containing ammonium formate (5 mM), pH 3
	Solvent B: acetonitrile:water (95:5) containing ammonium formate (5 mM), pH 3
	Gradient B (%): 0–4 min, 100%; 4–8 min, 100 to 60% linear gradient; 8–12 min, 60%; 12.1–17 min, 100%
	Flow rate (mL min^{-1}): 0.2 (12.1–15 min; 0.4)
	Column temp. (°C): 30
	Injection volume (μL): 2
	Sample solvent: acetonitrile:water (95:5)
MMLC	*Column:* Thermo Acclaim™ Trinity P1 (2.1 mm × 100 mm, 3 μm)
	Solvent A: 20 mM ammonium acetate in water, pH 5
	Solvent B: acetonitrile
	Gradient B (%): 0–2 min, 60%; 2–10.5 min, 60 to 80% linear gradient; 10.5–20.5 min, 80%; 21–34.5 min, 20%; 35–45 min, 60%
	Flow rate (mL min^{-1}): 0.2

	Column temp. (°C): 30
	Injection volume (μL): 5
	Sample solvent: acetonitrile:water (3:2)
RPLC	*Column:* Waters ACQUITY UPLC HSS T3 (2.1 mm × 100 mm, 1.8 μm)
	Solvent A: 5 mM ammonium formate in water
	Solvent B: 5 mM ammonium formate in methanol
	Gradient B (%): 0–1 min, 0%; 1–5 min, 0 to 100% linear gradient; 5–8.2 min, 100%; 8.3–12 min, 0%
	Flow rate (mL min^{-1}): 0.5
	Column temp. (°C): 30
	Injection volume (μL): 10
	Sample solvent: water

Table S3 Experimental conditions used to evaluate the six SPE cartridges[a]

SPE cartridge<br>Stationary phase	WCX<br>Mixed-mode	MCX<br>Mixed-mode	HLB, PS2<br>Reversed-phase	ENVI-Carb<br>Carbon	AC2<br>Activated carbon		
					Protocol 1	Protocol 2	Protocol 3
Conditioning 1	NH_3:MeOH (5:95) 5 mL	MeOH 5 mL	DCM 5 mL	DCM 5 mL	DCM 5 mL	ACN 10 mL	ACN 10 mL
Conditioning 2	H_2O 5 mL	H_2O 5 mL	MeOH 5 mL	MeOH 5 mL	MeOH 5 mL	H_2O 10 mL	H_2O 10 mL
Conditioning 3			Ace 5 mL	Ace 5 mL	Ace 5 mL		
Conditioning 4			H_2O 5 mL	H_2O 5 mL	H_2O 5 mL		
Wash	H_2O 5 mL	2 vol% FA in H_2O 5 mL	H_2O 5 mL	H_2O 5 mL	H_2O 5 mL	H_2O 10 mL	H_2O 10 mL
Elution fr. 1	2 vol% FA in MeOH 10 mL	MeOH 10 mL	Ace 10 mL	Ace 10 mL	Ace 10 mL	ACN:MeOH (3:2) 0–10 mL	ACN: H_2O (9:1) 10 mL
Elution fr. 2		NH_3:MeOH (5:95) 10 mL	MeOH 10 mL	MeOH 10 mL	MeOH 10 mL	ACN:MeOH (3:2) 10–20 mL	
Elution fr. 3			DCM 10 mL	DCM 10 mL	DCM 10 mL	ACN:MeOH (3:2)20–30 mL	
Elution fr. 4				2 vol% FA in MeOH:DCM (20:80) 10 mL	2 vol% FA in MeOH:DCM (20:80) 10 mL		

[a]Ace: acetone, ACN: acetonitrile, DCM: dichloromethane, MeOH: methanol, FA: formic acid, NH_3: 25 wt% aqueous ammonia solution, fr.: fraction

Table S4 Multiple reaction monitoring (MRM) transitions of target analytes

Analytes	Precursor ion *m/z*	Product ion *m/z*	Surrogate
DPG	212.1 > 119.1	212.1 > 94.0	DPG-d_{10}
DTG	240.2 > 133.0	240.2 > 108.0	DPG-d_{10}
TPG	288.2 > 92.1	288.2 > 195.1	DPG-d_{10}
TBG	192.2 > 60.0	192.2 > 133.0	DPG-d_{10}
CG	85.0 > 68.0	85.0 > 43.0	CG-$^{15}N_4$
CPG	161.1 > 102.0	161.1 > 144.1	DPG-d_{10}
HCG	251.2 > 209.3		DPG-d_{10}
DPG-d_{10}	222.2 > 124.1	222.2 > 99.1	
CG-$^{15}N_4$	89.0 > 71.0	89.0 > 45.1	

Table S5 LC-MS/MS measurement conditions

Parameters	Details
Equipment	Waters ACQUITY UPLC/ Waters Xevo TQ
LC column	MACHEREY-NAGEL NUCLEODUR HILIC (2.1 mm × 150 mm, 3 μm)
Mobile phase	Solvent A: acetonitrile:water (5:95) containing 5 mM ammonium formate, pH 3<br>Solvent B: acetonitrile:water (95:5) containing 5 mM ammonium formate, pH 3
Gradient B (%)	0–4 min, 100%; 4–8 min, 100 to 60% linear gradient; 8–12 min, 60%; 12.1–30 min, 100%
Flow rate (mL min^{-1})	0.2 (12.1–25 min; 0.4)
Injection volume (μL)	2
Ionization	ESI-positive
Source temp. (°C)	150
Column temp. (°C)	30
Capillary voltage (kV)	0.5
Cone gas flow (L h^{-1})	50
Desolvation temp. (°C)	600
Desolvation gas flow (L h^{-1})	1000

Table S6 Sample description

	Name	Sampling date	Sample type	Depth	Location	Connections between the samples
Used for QA/QC	Yodo River	11.05.2021	Surface water, river	-	Osaka Pref. (Japan)	-
Field sampling	LW-1	15.07.2021	Surface water, lake	0 m	Shiga Pref. (Japan)	LW-1 is the water resource of TW-1.
	LW-2	15.07.2021	Lake water around thermocline	30 m	Shiga Pref. (Japan)	LW-2 is obtained from the same location as that of LW-1.
	LW-3	15.07.2021	Bottom water, lake	50 m	Shiga Pref. (Japan)	LW-3 is obtained from the same location as that of LW-1.
	RW-1	06.07.2021	Surface water, river	-	Kyoto Pref. (Japan)	
	RW -2	06.07.2021	Surface water, river	-	Kyoto Pref. (Japan)	RW-2 is obtained downstream from LW-1.
	RW -3	06.07.2021	Surface water, river	-	Kyoto Pref. (Japan)	
	RW -4	06.07.2021	Surface water, river	-	Osaka Pref. (Japan)	Three rivers (RW-1, 2, and 3) flow downstream to join the river of RW-4. The downstream of RW-4 is the water resource of TW-2.
	SE-1	06.07.2021	Sewage effluent	-	Kyoto Pref. (Japan)	SE-1 flows into the upper stream of RW-2.
	SE-2	06.07.2021	Sewage effluent	-	Kyoto Pref. (Japan)	SE-2 flows into the upper stream of RW-2.
	SE-3	06.07.2021	Sewage effluent	-	Kyoto Pref. (Japan)	SE-3 flows into the upper stream of RW-3.
	SE-4	06.07.2021	Sewage effluent	-	Kyoto Pref. (Japan)	SE-4 flows into the upper stream of RW-3.

SE-5	06.07.2021	Sewage effluent	-	Kyoto Pref. (Japan)	SE-5 flows into the downstream of RW-3.
TW-1	15.07.2021	Tap water	-	Shiga Pref. (Japan)	TW-1 is drinking water originated from LW-1.
TW-2	06.07.2021	Tap water	-	Osaka Pref. (Japan)	TW-2 is drinking water originated from the downstream of RW-4.

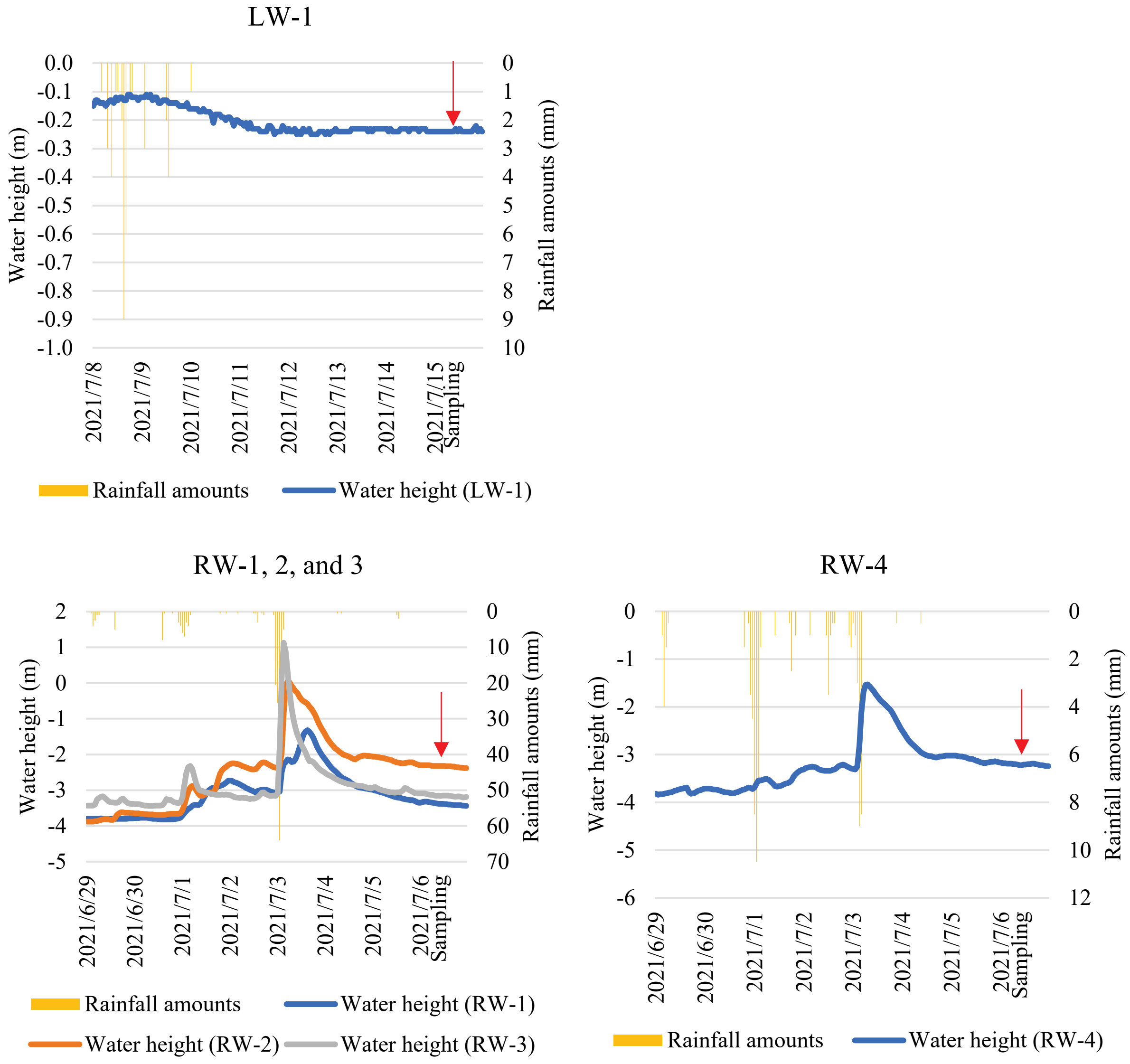

Fig. S1 Rainfall amount and water height at each sampling site (lake and river) prior to sampling

Rainfall amounts were obtained from areas close to each sampling point. The water height values of LW-1 were obtained from observation points near the sampling points, whereas those of RW-1–4 were obtained from the sampling sites. The red arrows in each figure indicate the sampling date and time.

Examination of water sampling methods

When a Van Dorn sampler with a rubber part (RIGO, Japan) was used to sample lake water in our preliminary investigation, DPG was detected at higher concentrations (35–60 ng L^{-1}) than was detected in river water (7.0–9.1 ng L^{-1}) and sewage effluent (6.5–30 ng L^{-1}). Therefore, other water sampling methods were investigated to verify DPG contamination from the Van Dorn sampler. A stainless steel bucket, a RIGO-B transparent water bottle (main materials: acrylic resin, polycarbonate, and polyvinyl chloride; RIGO, Japan), and the Van Dorn sampler were used to sample lake surface water (n = 1), and the DPG concentrations in each were compared (**Fig. S2**). Only DPG and CG were detected in these samples. The DPG concentrations in the water sampled using the stainless steel bucket and RIGO-B transparent water bottle were comparable (~5 ng L^{-1}), whereas that in the water sampled using the Van Dorn sampler was 58 times higher (290 ng L^{-1}). The sampler type had no effect on the CG concentration, which implied that DPG was the only contaminant among the target analytes. RIGO-B transparent water bottles and Van Dorn samplers are typically used to sample water at different depths. The former sampler does not contain rubber, whereas the latter contains rubber plugs at either end. DPG is used as a vulcanization accelerator to manufacture rubber products. The sampled water was significantly contaminated with DPG in a few minutes after coming into contact with the rubber parts of the Van Dorn sampler, indicating that the use of rubber-containing products causes DPG contamination. Hence, the RIGO-B transparent water bottle was used for subsequent sampling. Furthermore, contact with rubber products during sample preparation was eliminated.

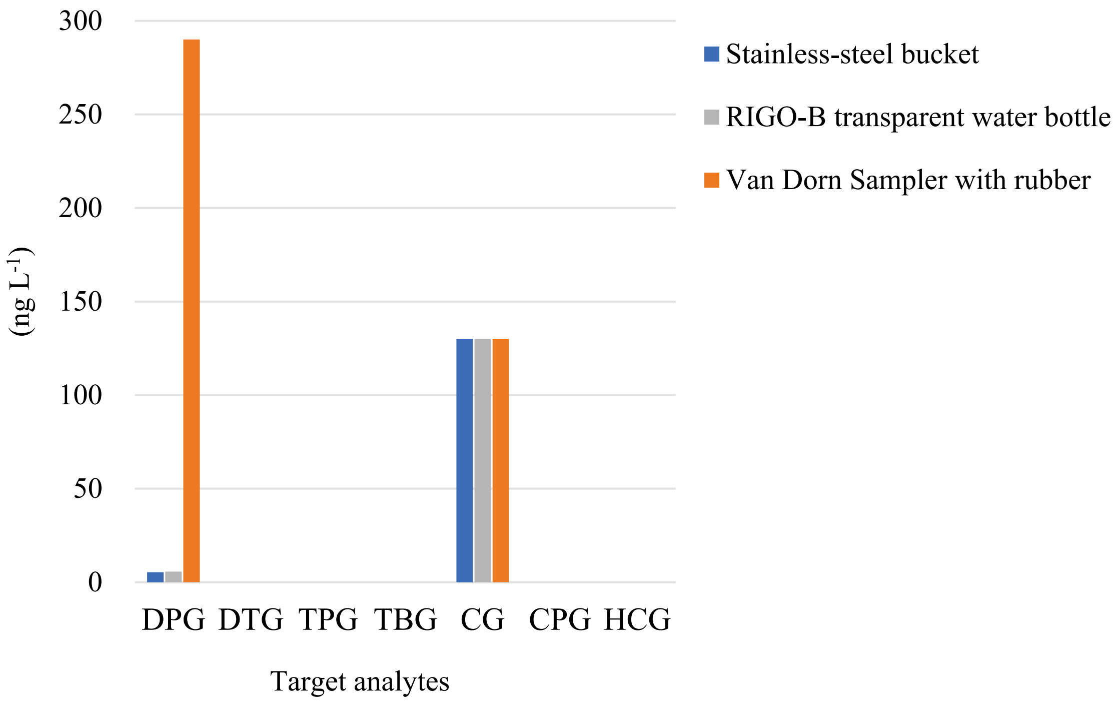

Fig. S2 DPG concentrations determined in lake surface water sampled using different devices

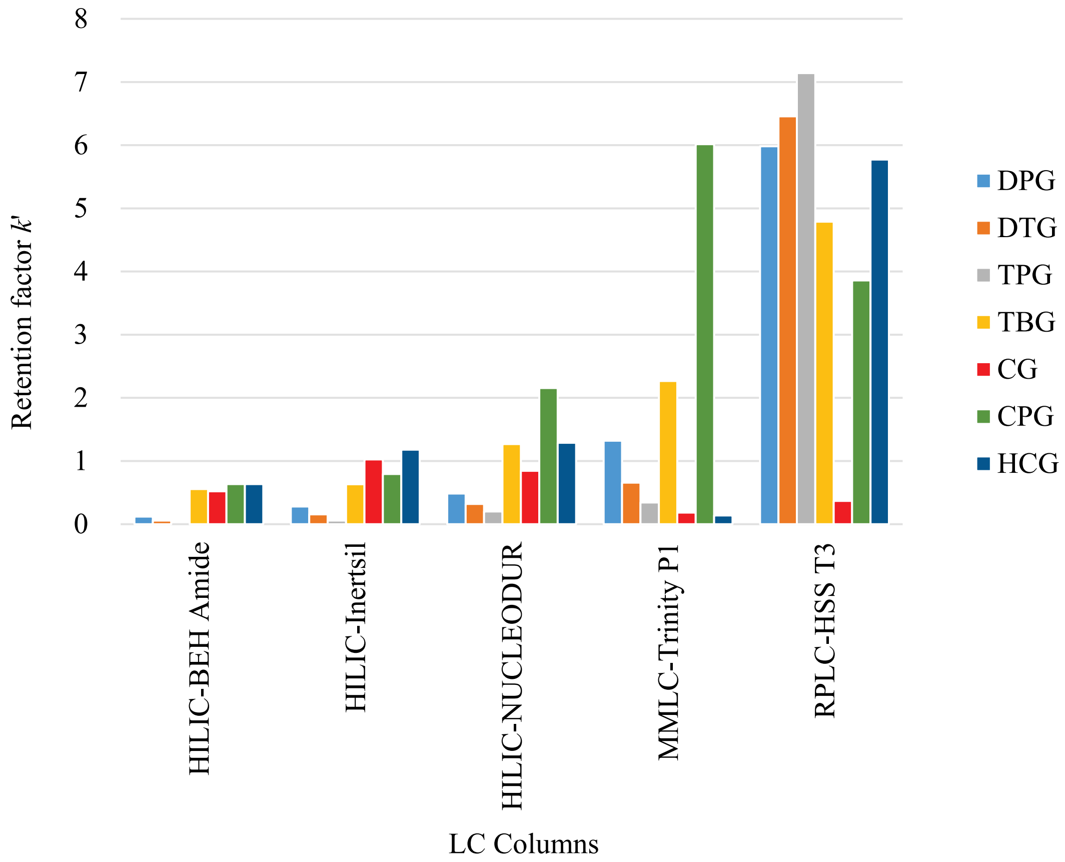

Fig. S3 Retention factors (*k*′) of target analytes obtained for the five examined LC columns

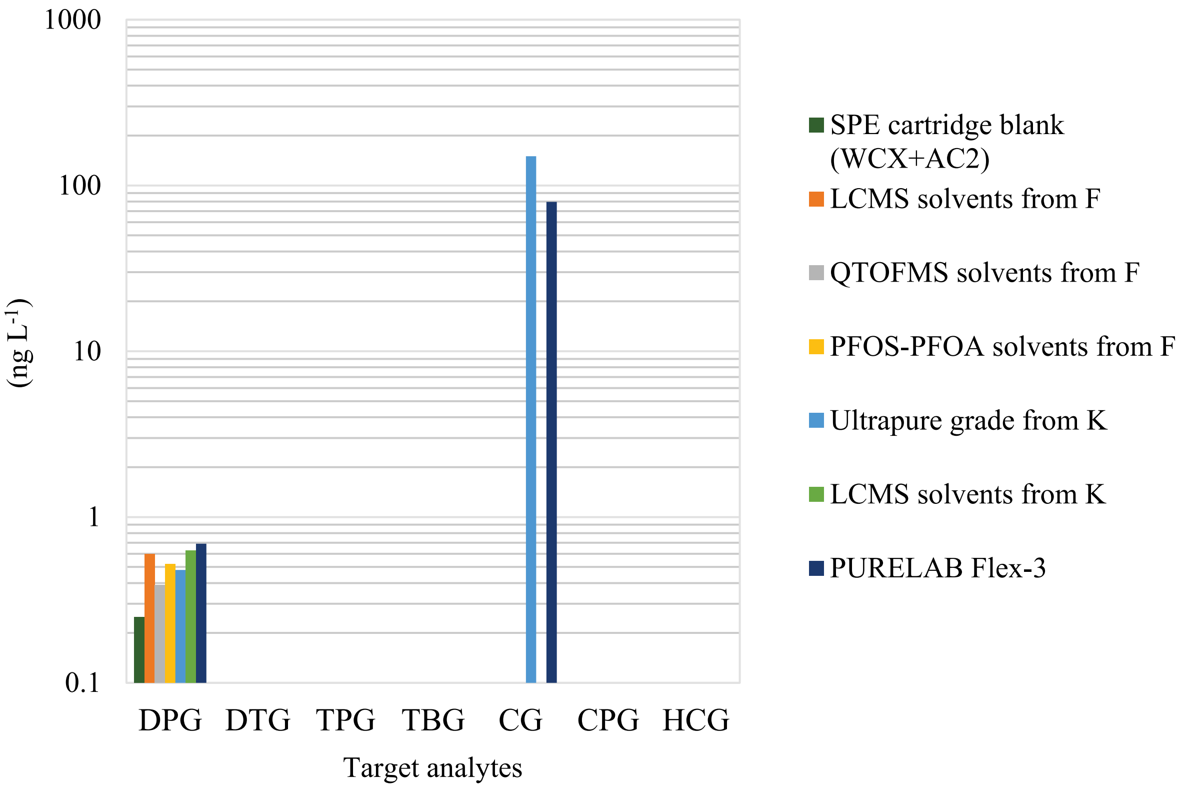

Fig. S4 Blank test results of SPE cartridges and various ultrapure water samples

Ultrapure water was purchased from Fujifilm Wako Pure Chemical Corporation (Osaka, Japan) (denoted as F) and Kanto Chemical Co., Inc. (Tokyo, Japan) (denoted as K). PURELAB Flex-3 denotes ultrapure water obtained in-house using the corresponding purification system.

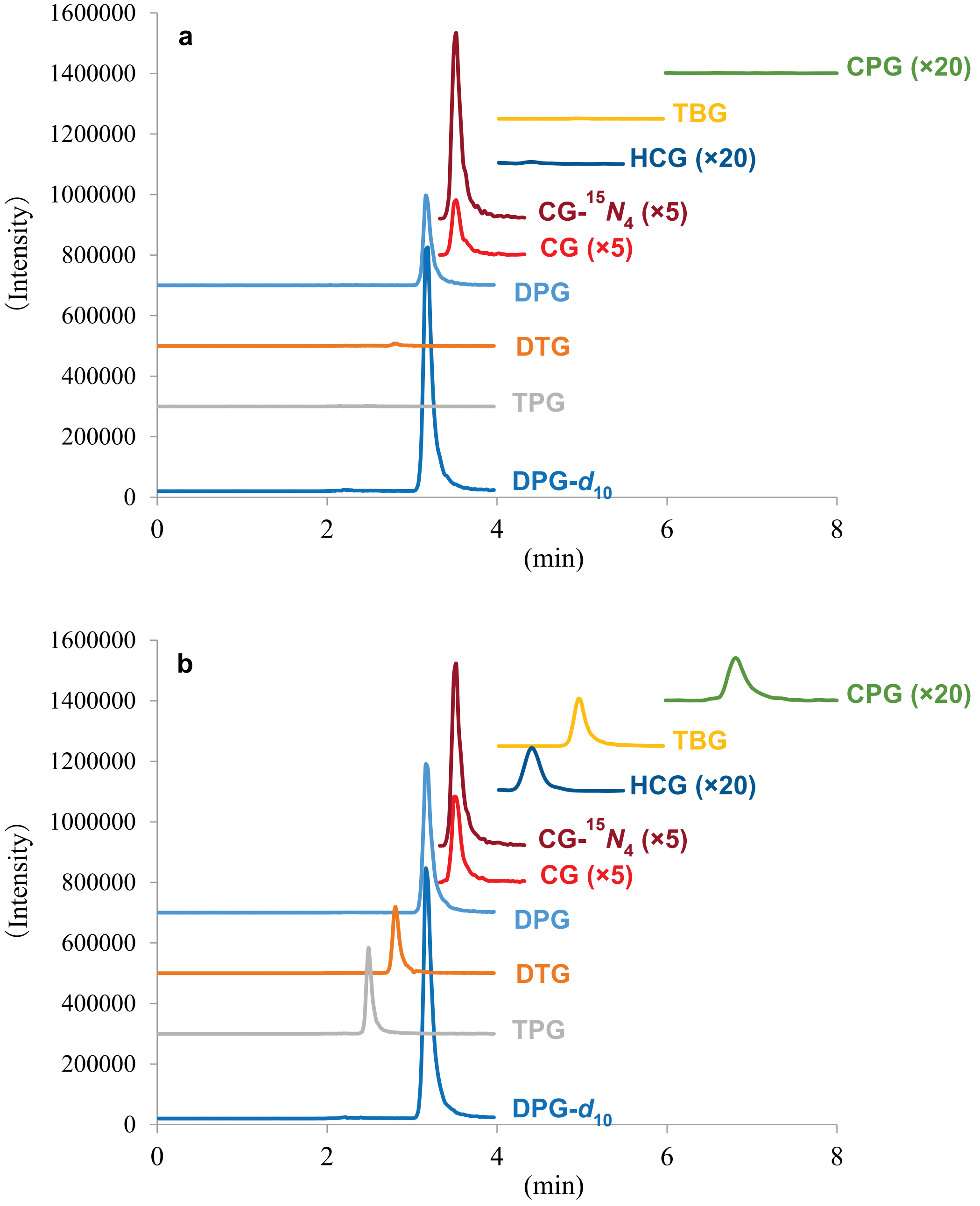

Fig. S5 Chromatograms of target analytes in the method validation

The sample descriptions were as follows: **a** Non-spiked Yodo River water sample, **b** Standard-spiked Yodo River water sample. Chromatograms of CG and CG-$^{15}N_4$ are plotted with five-fold intensities and those of CPG and HCG are plotted with 20-fold intensities.
